# Two-Stage Seebeck Effect in Charged Colloidal Suspensions

**DOI:** 10.3390/e23020150

**Published:** 2021-01-26

**Authors:** Ioulia Chikina, Sawako Nakamae, Valeriy Shikin, Andrey Varlamov

**Affiliations:** 1 LIONS, NIMBE, CEA, CNRS, Université Paris-Saclay, CEA Saclay, 91191 Gif-sur-Yvette, France; julia.chikina@cea.fr; 2 Service de Physique de l’etat Condensé, SPEC, CEA, CNRS, Université Paris-Saclay, CEA Saclay, 91191 Gif-sur-Yvette, France; sawako.nakamae@cea.fr; 3 ISSP, RAS, Chernogolovka, 142432 Moscow, Russia; shikin@issp.ac.ru; 4 CNR-SPIN, c/o DICII-Università di Roma Tor Vergata, Via Del Politecnico 1, 00133 Roma, Italy

**Keywords:** seebeck effect, colloids, thermodiffusion

## Abstract

We discuss the peculiarities of the Seebeck effect in stabilized electrolytes containing the colloidal particles. Its unusual feature is the two stage character, with the linear increase of differential thermopower as the function of colloidal particles concentration $n_{⊙}$ during the first stage (“initial state”) and dramatic drop of it at small $n_{⊙}$ during the second one (“steady state”). We show that the properties of the initial state are governed by the thermo-diffusion flows of the mobile ions of the stabilizing electrolyte medium itself and how the colloidal particles participate in the formation of the electric field in the bulk of the suspension. In its turn, we attribute the specifics of the steady state thermoelectric effect the massive colloidal particles undergoing slow thermal diffusion and the break down of their electro-neutrality in the vicinity of electrodes.

## 1. Introduction

In recent years, liquid thermoelectric materials are emerging as a cheaper alternative to the semiconductor-based solid counterpart for low-grade waste heat recovery technologies. A breakthrough in the enhancement of the thermoelectric efficiency of thermo-electrochemical cell (hereafter called “thermocel”) has been achieved using ionic liquids [1]. More recently, the dispersion of charged colloidal particles (magnetic nanoparticle) was also found to increase the Seebeck coefficient of the host electrolyte. Incorporation of nanometer-sized colloidal particles can considerably change the transport properties of such systems. For example, in Ref. [2] a novel use of charged colloidal solution was proposed to improve the Seebeck coefficient of an aqueous thermocell. The authors study transport properties of the charged colloidal suspensions of iron oxide nanoparticles (maghemite) dispersed in aqueous medium and report the values of the order of 1–1.5 mV/K for the Seebeck coefficient. The inclusion of tetrabutyl ammonium as counterions, lead to an enhancement of the fluid’s initial Seebeck coefficient by 15% (at nanoparticle volume concentration 1%). The authors of Ref. [3] also indicate high Seebeck coefficient values (≈2 mV/K) for many electrolyte–electrode combinations, exceeding existing predictions.

In point of fact, when the colloidal particles are neutral, they cannot exist independent in a dilute solution. Rather, they coagulate due to the van der Waals forces acting between them. In order to prevent such coagulation processes, one can immerse individual colloidal particles in the electrolyte specific for each sort of them such that they acquire surface ions (e.g., hydroxyl groups, citrate, etc. [4,5,6]) resulting in a very large structural charge eZ (|Z|≫10). Its sign can be either positive and negative, depending on the surface group type. Such a procedure is called stabilization and obtained suspension is considered stabilized.

**Figure 1:**
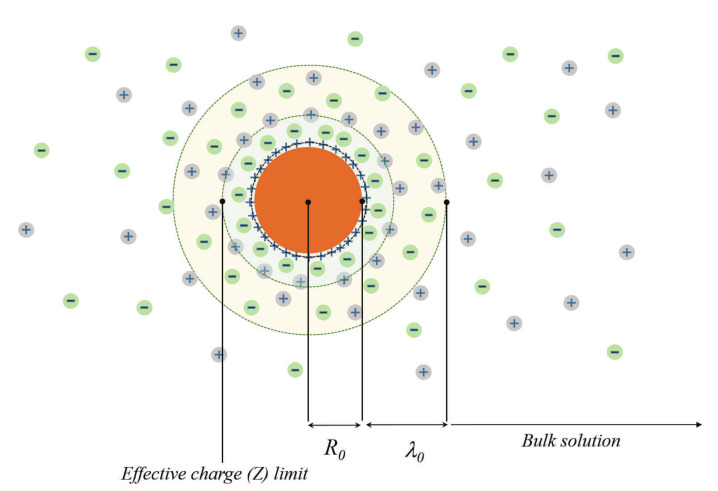
The schematic presentation of the multiply-charged colloidal particle surrounded by the cloud of counter-ions.

The large structural charge attracts counterions from the surrounding solvent creating an electrostatic screening coat of the length $\lambda_{0}$ with an effective charge −eZ (see Figure 1). In these conditions, nanoparticles approaching within the distances $r\leq \lambda_{0}$ between them begin to repel each other preventing coagulation. The corresponding theory of stabilized electrolyte was developed in Refs. [7,8,9] and is often referred to as the DLVO theory.

A clear manifestation of the stabilization phenomenon occurs in such diluted solutions especially in the region of concentrations where

(1) $$n_{⊙}{\left(\lambda_{0}+R_{0}\right)}^{3}\ll 1,$$

where $n_{⊙}$ is the density of colloidal particles and $R_{0}$ is the bare radius of the colloidal particle. It is important to note that the stabilized DLVO solution is homogeneous under the condition described in Equation (1).

When a temperature gradient is applied across a thermocell, three types of thermodiffusion flows arise: two of them correspond to the motion of the ions of different signs and mobilities $\mu_{\pm }$, belonging to a stabilizing electrolyte, while the third one is related to the diffusion of less-mobile colloidal particles ($\mu_{⊙}\ll \mu_{\pm }$). At the first stage (“initial state”), the displacement of electrolyte ions leads to the formation of the conventional Seebeck field proportional to $\left(\mu_{+}-\mu_{-}\right)$ [10,11,12,13,14]. As time passes, the drag of the less-mobile colloidal particles by the heat flow results in appearance of a particle concentration gradient (Soret effect). After considerable time from the beginning of the experiment (due to a very large difference in the mobility of colloids and ions of the stabilizing electrolyte: $\mu_{⊙}\ll \mu_{\pm }$) their redistribution along the length of the cell becomes essential. The second stage “steady state” sets in: the presence of colloidal particles, in accordance to the data of Ref. [2], starts to dramatically affect the Seebeck coefficient values.

The characteristic feature of the steady state is the appearance of specific electrostatic forces acting on colloidal particles approaching the vicinity of the edge of the thermocell; i.e., the electrodes (see [15,16]). In fact, the accumulation layer had already been formed there during the initial state due to the fast thermodiffusion of the light ions. When colloidal particles approach the electrodes at distances of the order of Debye length, the particles lose their electro-neutrality and begin to be swallowed into the accumulation layer, which modifies the value of their surface charges. As it will be demonstrated below, this effect is important for the description of the steady stage at the relatively low concentrations of colloidal particles. Let us mention that these electrostatic forces are specific to the edge (electrode/liquid interface) region, decoupled from the thermodiffusion dynamics in the bulk solution only and there is no need to consider them in traditional thermophoretic study of the dynamics of the colloidal particle in the bulk of the electrolyte (see [13,14,17,18,19]).

In this work we study the effect of presence of colloidal particles in stabilizing electrolyte on its thermoelectric properties and give an explanation to the above described peculiarities observed in experiments. The paper is organized as follows. In the second section, we study the properties of the initial stage of the process occurring in the thermocell under an imposed temperature gradient, when the mobile ions of the stabilizing electrolyte diffuse, while the displacement of the colloidal particles is still inessential. The third section is devoted to the analysis of the peculiarities of the steady stage, when considerable displacements of the massive colloidal particles induced by the slow thermal diffusion results in the breakdown of their electro-neutrality in the vicinity of the electrodes. The last section summarizes the results obtained and sheds light upon the discrepancy between the steady-state establishment time lapse in the experiment and the existing theoretical estimation.

## 2. Initial State of Seebeck Effect in Colloidal Solution

In order to understand the impact of colloidal particles on the Seebeck signal of a suspension at the initial state, let us assume that the colloidal particles remain immobile, i.e., their concentration $n_{⊙}$ can be considered homogeneous within the experimental time-scale corresponding to this stage.

The electric current, **j**, in the conducting media in the presence of electric field **E** and a temperature gradient **∇ T** is described by the generic equation [10,11,12,13,14]

(2) j=σE−β∇T.

In Equation (2) β=−Sσ and σ is electrical conductivity while *S* is the Seebeck coefficient (also known as thermopower). For further convenience we express the former in terms of σ and *S*, namely, the Seebeck coefficient determines the voltage appearing across the thermocell in open-circuit related to the applied temperature difference

(3) $$V=\int_{T_{1}}^{T_{2}}S\left(T\right)dT.$$

Since the electrolyte consists of two oppositely charged subsystems of positive and negative ions, its effective Seebeck coefficient is determined by the sum of the coefficients $\beta_{\pm }$ of each ion subsystem divided by the total conductivity ($\sigma_{+}+\sigma_{-}$) of the solution:

(4) $$S_{\mathrm{tot}}=-\frac{\beta_{+}+\beta_{-}}{\sigma_{+}+\sigma_{-}}=\frac{S_{-}\sigma_{-}+S_{+}\sigma_{+}}{\sigma_{-}+\sigma_{+}}.$$

The conductivity of an electrolyte with dilute concentration (see condition (1)) of colloidal particles was recently studied in Ref. [20], where the explicit expression for it was obtained as,

(5) $$\begin{array}{ccc} \sigma_{\mathrm{tot}}\left(n_{⊙}\right) & = & \sigma_{+}\left(n_{⊙}\right)+\sigma_{-}\left(n_{⊙}\right),\\ \sigma_{\pm }\left(n_{⊙}\right)\; & = & \;\sigma_{\pm }^{\left(0\right)}\;\left[1\;+\;4\pi n_{⊙}\left(\frac{\gamma_{\pm }-1}{\gamma_{\pm }+2}\right){\left(R_{0}+\lambda_{0}\right)}^{3}\right], \end{array}$$

where $\sigma_{\pm }^{\left(0\right)}$ are the conductivities of the ion subsystems in the absence of colloidal particles, $\gamma_{\pm }=\sigma_{⊙}^{\pm }/\sigma_{\pm }^{\left(0\right)}$ and $\sigma_{⊙}^{\pm }$ is the effective conductivity of the ions in the screening coat of the colloidal particles with the corresponding signs. For a negatively charged screening coat, the positive ions are drawn towards the particle surface and thus, $\gamma_{+}>1$. Vice-versa, the conductivity of the negative ions is suppressed due to the repulsion by the screening coats, resulting in $\gamma_{-}<1$. Combined, the effect of colloidal particles on the positive ions is dominant.

The conductivity growth as the colloidal particles are introduced into the electrolyte can be understood as the facilitation of charge transfer in the medium where some fraction of the volume is occupied by these highly conducting objects. As a result, under the same intensity of electric field E current increases. The situation is different for the Seebeck coefficient *S*. Indeed, *S* characterizes the voltage response of the medium to the applied temperature gradient and thus there is no obvious reason to suppose direct dependence of $S_{\pm }$ on the colloidal particles concentration.

In this assumption Equation (4) acquires the form

(6) $$S_{\mathrm{tot}}\left(n_{⊙}\right)=S_{\mathrm{tot}}^{\left(0\right)}+\Delta S\left(n_{⊙}\right),$$

where

(7) $$S_{\mathrm{tot}}^{\left(0\right)}=\frac{S_{-}^{\left(0\right)}\sigma_{-}^{\left(0\right)}+S_{+}^{\left(0\right)}\sigma_{+}^{\left(0\right)}}{\sigma_{-}^{\left(0\right)}+\sigma_{+}^{\left(0\right)}},$$

and

(8) $$\begin{array}{cc} \Delta S_{\mathrm{tot}}\left(n_{⊙}\right)= & \frac{12\pi n_{⊙}\left(\gamma_{+}-\gamma_{-}\right){\left(R_{0}\;+\;\lambda_{0}\right)}^{3}}{\left(\gamma_{+}+2\right)\left(\gamma_{-}+2\right)}\times \\ & \frac{\sigma_{-}^{\left(0\right)}\sigma_{+}^{\left(0\right)}\left(S_{+}^{\left(0\right)}-S_{-}^{\left(0\right)}\right)}{{\left(\sigma_{-}^{\left(0\right)}+\sigma_{+}^{\left(0\right)}\right)}^{2}}. \end{array}$$

Normalized change in the Seebeck coefficient as a function of the colloidal particle concentration (as reported in Ref. [2]) takes the form:

(9) $$\begin{array}{c} \frac{\Delta S_{\mathrm{tot}}\left(n_{⊙}\right)}{S_{\mathrm{tot}}^{\left(0\right)}}=12\pi n_{⊙}\frac{\left(\gamma_{+}-\gamma_{-}\right){\left(R_{0}\;+\;\lambda_{0}\right)}^{3}}{\left(\gamma_{+}+2\right)\left(\gamma_{-}+2\right)}\times \\ \frac{\left(S_{+}^{\left(0\right)}-S_{-}^{\left(0\right)}\right)}{S_{-}^{\left(0\right)}\left(1+\sigma_{-}^{\left(0\right)}/\sigma_{+}^{\left(0\right)}\right)+S_{+}^{\left(0\right)}\left(1+\sigma_{+}^{\left(0\right)}/\sigma_{-}^{\left(0\right)}\right)}.\; \end{array}$$

**Figure 2:**
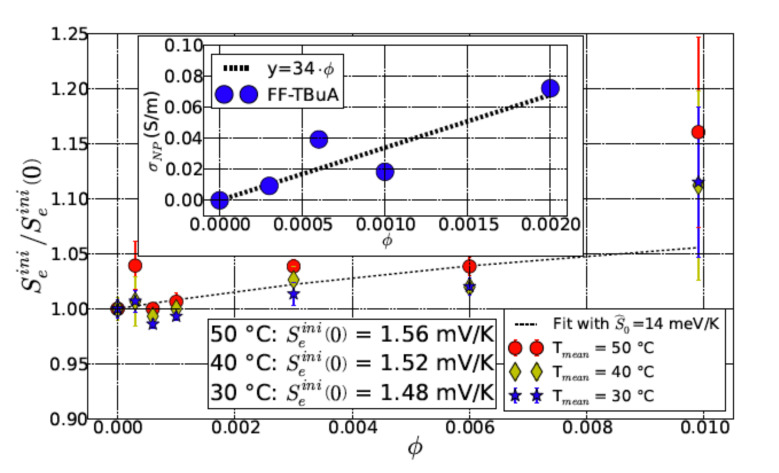
Normalized initial state Seebeck coefficient and electrical conductivity (inset) as a function of nanoparticles concentration (For reproduction of material from PCCP: reproduced from Ref. [2] with permission from the PCCP Owner Societies).

It should be noted here that the thermogalvanic contribution to the overall temperature coefficient (ΔV/ΔT) [21] is not taken into consideration. This is justified because this term is additive to $S_{\mathrm{tot}}^{\left(0\right)}$ and is independent of the nanoparticle concentration [2] and thus does not interfere with the $\Delta S_{\mathrm{tot}}^{\left(0\right)}$ in the Equation (9). The obtained result convincingly demonstrates that the Seebeck coefficient follows the linear growth of the colloidal particles concentration in the considered range (*cf* Equation (1)) as observed experimentally (see Figure 2). This linear growth differs from that of the conductivity $\Delta \sigma_{\mathrm{tot}}\left(n_{⊙}\right)$ (see Equation (5)) which is proportional to the sum of $\sigma_{-}^{\left(0\right)}$ and $\sigma_{+}^{\left(0\right)}$. Here, $\Delta S_{tot}$ is proportional to the difference $S_{+}^{\left(0\right)}\;-\;S_{-}^{\left(0\right)}$ of the formal ion Seebeck coefficients in the absence of colloidal particles. Such a result is very natural: Seebeck effect always (in metals, in semiconductors) is related to the dissimilarity of the charge carriers. Hence, the direct measurements of $\sigma_{\mathrm{tot}}\left(n_{⊙}\right)$ and $S_{\mathrm{tot}}\left(n_{⊙}\right)$ along with the independent knowledge of $\gamma_{\pm }$ and $R_{0}+\lambda_{0}$ (according to Refs. [2,20] $\lambda_{0}\approx 60Å,R_{0}\approx 70Å$) allow us to determine the values of $\sigma_{\pm }^{\left(0\right)}$.

## 3. Steady State Seebeck Effect in Colloidal Solution

Application of a temperature difference across the thermocell results in the charge separation among electrolyte ions. This happens first due to the difference of their coefficients $\beta_{\pm }$, and second, due to the difference of their diffusion coefficients [22]. The latter’s contribution to the Seebeck coefficient is specific for semiconductors and electrolytes and accounts for the thermodiffusion of charged particles. As a result, the accumulated layers of ions of opposite charges are formed in the vicinity of electrodes at the initial state of the Seebeck effect.

**Figure 3:**
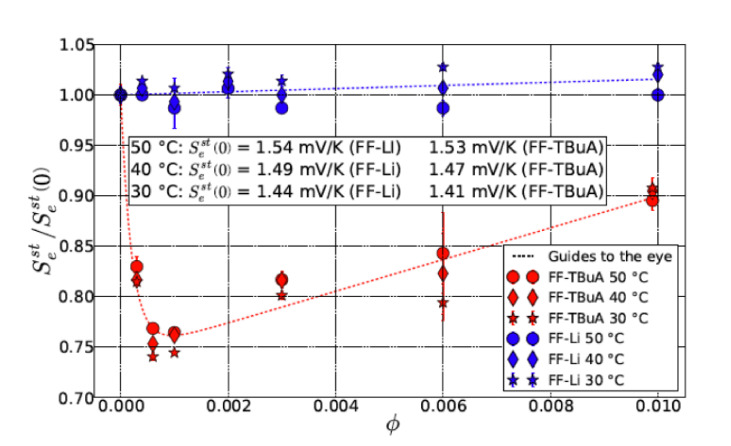
Normalized steady state Seebeck coefficient as a function of nanoparticles concentration (For reproduction of material from PCCP: reproduced from Ref. [2] with permission from the PCCP Owner Societies).

When an electrolyte contains a sufficiently small concentration of colloidal particles the measured value of the Seebeck coefficient decreases over time. This decrease as a function of particles concentration is drastic at the beginning, then the Seebeck coefficient reaches a minimum and finally, it grows linearly in accordance with Equation (9) (see Figure 3) [2].

One of the reasons of its occurrence can be the complex structure of the colloidal particles surrounded by their screening coatings. The process of thermodiffusion results in their slow drift whose direction depends sensitively on the ionic environment surrounding the colloidal particles. It has been known for a while now that in some colloidal suspensions the particles move toward the cold, and in others toward the warm region, depending on the resepective sign of their Soret coefficient [17,18,19,23,24,25]. Let us recall, that in Ref. [20] the colloidal particle screening was considered in the spherically symmetrical situation by means of solution to the Poisson equation with the zero boundary conditions at infinity. Close to the electrode the Seebeck electric field in the electrolyte is formed mainly due to the redistribution of ions and becomes non-homogeneous [26]. As a consequence, the colloidal complexes, acquiring induced dipole moments, are pulled into the region of stronger electric field.

In the vicinity of the electrodes, the difference in electrostatic attraction forces on the charged counterions of the screening coat and the colloidal particle core to the metallic electrode enters in play. Indeed, such attraction forces are very different for the weakly charged counterions in the coating layer and the strongly charged (Q=Ze) particle core of the colloid. As a result, the particle-coat clusters lose their electro-neutrality and start compensating the charge of accumulative ion layer at the electrode surface.

Let us evaluate the value of particles concentration corresponding to the minimum in the Seebeck coefficient at Figure 3. For this purpose we recall the electrostatics problem of a point charge in interaction with the conducting plane, separating two semi-spaces each with a dielectric constant of $\epsilon_{1}$ and $\epsilon_{2}$. This situation can be reduced to that of the charge interacting with the corresponding electrostatic image charge behind the plane (see Ref. [27]):

(10) $$F_{\epsilon }\left(z\right)=\frac{Q^{2}\left(\epsilon_{1}-\epsilon_{2}\right)}{4\epsilon_{1}\left(\epsilon_{1}+\epsilon_{2}\right)z^{2}}.$$

In derivation of Equation (10) the charge was supposed to be placed at the distance *z* from the plane in the semi-space with a dielectric constant $\epsilon_{1}$.

When the semi-space is filled by an electrolyte, the electrostatic image force $F_{e}$ (Equation (10)) is screened at the distances of the order of the Debye length from the plane as demonstrated by Wagner and Onsager (Refs. [15,16]):

(11) $$F_{\mathrm{WO}}\left(z\right)=F_{\epsilon }\left(z\right)\exp\left(-\frac{2z}{\lambda_{0}}\right).$$

with a corresponding electrostatic energy of

$$U_{\mathrm{WO}}\left(z\right)\;=\;-\;\int_{z}^{\infty }\;\;F_{\mathrm{WO}}\left(x\right)dx\;=\;\frac{Q^{2}\left(\epsilon_{1}\;-\;\epsilon_{2}\right)}{2\lambda_{0}\epsilon_{1}\left(\epsilon_{1}\;+\;\epsilon_{2}\right)}\Gamma \left(\;-1,\frac{2z}{\lambda_{0}}\right),$$

where $\Gamma \left(s,x\right)$ is the upper incomplete gamma function. In other words, a charged particle located in the electrolyte at distances exceeding the Debye length $\lambda_{0}$ from the electrode interacts exponentially weakly with it. In the case under consideration here, we assume the dielectric constant of the metallic electrode $\epsilon_{2}\to \infty$, while $\epsilon_{1}=\epsilon_{aq}$. The corresponding electrostatic energy of the colloidal particle core is

(12) $$U_{\mathrm{WO}}\left(z\right)=-\frac{Z^{2}e^{2}}{2\lambda_{0}\epsilon_{aq}}\Gamma \left(-1,\frac{2z}{\lambda_{0}}\right).$$

**Figure 4:**
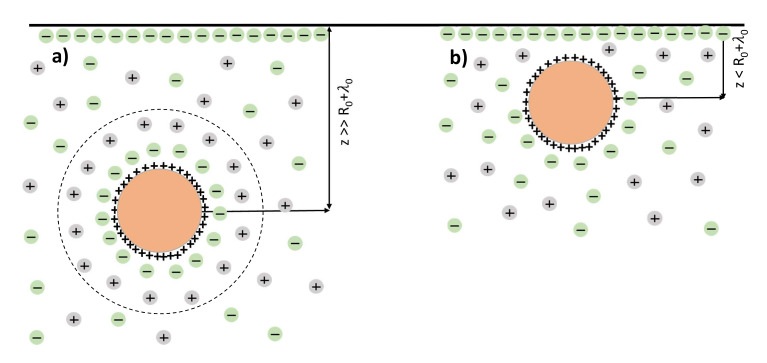
Colloidal particles in an stabilizing electrolyte. (**a**) Until the colloidal particle size exceeds the Debye length it keeps its integrity. (**b**) When the colloidal particle approaches the charged electrode at the distances less than its size the cluster loses its electro-neutrality.

The effective radius of the colloidal particle is the sum of the radius of charged core and the thickness of the screening coat: $R_{0}+\lambda_{0}$. Hence until the particle–electrode distance exceeds the Debye length, the colloidal particle keeps its integrity (see Figure 4a) and the core–electrode interaction energy can be determined by Equation (12). On the contrary when the colloidal particle approaches the charged electrode at distances less than its size it loses its electro-neutrality (see Figure 4b) and the framework of the Poisson equation with infinite boundary conditions [20] is no longer applicable. The detailed study of the electrostatic interactions between a planar surface and a charged sphere immersed in the electrolyte media was performed by Ohshima in Ref. [28]. The author found the corresponding interaction energy $U_{\mathrm{HO}}$ as

(13) $$U_{\mathrm{HO}}\left(z\right)=U_{0}exp\left(-\frac{z-R_{0}}{\lambda_{0}}\right).$$

Applying this consideration to the case of the colloidal particle core and matching Equations (12) amd (13) at the distance $z=R_{0}+\lambda_{0}$ one can find an energy constant $U_{0}$:

$$U_{0}∼-\frac{Z^{2}e^{2}}{\lambda_{0}\epsilon_{aq}}\Gamma \left(-1,2+\frac{2R_{0}}{\lambda_{0}}\right).$$

Now one can estimate the maximal colloidal particles surface concentration $N_{⊙}^{max}$ which can be localized in the vicinity of the electrode surface through the mechanism discussed above. Their attraction to the electrode, charged due to the presence of excess ions with the surface concentration $N_{-}$, continues until the latter will not be compensated by the positive structural charges of the particle core:

(14) $$N_{⊙}^{max}=N_{-}/Z.$$

The value of $N_{-}$ can be found knowing the values of the Seebeck coefficient at the end of the initial state $S_{\mathrm{tot}}$, and the temperature gradient. Indeed, considering a thermocell with an electrolyte as a parallel plate capacitor, one can write:

$$E=\frac{4\pi eN_{-}}{\epsilon_{aq}},$$

from which,

(15) $$N_{-}=\frac{\epsilon_{aq}}{4\pi e}S_{\mathrm{tot}}\left(\frac{\Delta T}{\Delta L}\right).$$

The value of the particle concentration at the electrode surface $N_{⊙}$ can be found by comparing the homogeneous distribution of the colloidal particles in the bulk solution to that in the presence of an electrostatic potential of the electrode, determined by Equations (12) and (13):

(16) $$\begin{array}{c} N_{⊙}\;=\;n_{⊙}\left\{\int_{R_{0}}^{R_{0}+\lambda_{0}}\left(exp\left[\frac{Z^{2}e^{2}\Gamma \left(-1,2+\frac{2R_{0}}{\lambda_{0}}\right)}{\lambda_{0}k_{B}T\epsilon_{aq}}exp\left(-\frac{z-R_{0}}{\lambda_{0}}\right)\right]-1\right)dz\right.\\ \left.+\int_{R_{0}+\lambda_{0}}^{\infty }\left(exp\left[\frac{Z^{2}e^{2}}{2\lambda_{0}k_{B}T\epsilon_{aq}}\Gamma \left(-1,\frac{2z}{\lambda_{0}}\right)\right]-1\right)dz\right\}. \end{array}$$

Equation (16) relates the surface concentration of the colloidal particles $N_{⊙}$ to the volume concentration $n_{⊙}$ in the bulk electrolyte.

Using the asymptotic expressions for incomplete Gamma-function

$$\Gamma \left(-1,x\right)=\left\{\begin{array}{cc} e^{-x}/x^{2}, & x\gg 1\\ 1/x & x\ll 1 \end{array}\right.$$

and making sure that $Z^{2}e^{2}\ll \lambda_{0}k_{B}T\epsilon_{aq}$ (the characteristic values of the parameters $Z\approx 300,\epsilon_{aq}\approx 80$, $\lambda_{0}\approx 60\;Å,R_{0}\approx 70\;Å$) obtained from analyzing the data of Ref. [2] with the help of Ref. [20]), one can expand the exponents in Equation (16) to

(17) $$N_{⊙}∼n_{⊙}\frac{Z^{2}e^{2}}{k_{B}T\epsilon_{aq}}\frac{e^{-2\left(1+\frac{R_{0}}{\lambda_{0}}\right)}}{{\left(1+\frac{R_{0}}{\lambda_{0}}\right)}^{2}}.$$

In order to estimate the corresponding concentration values let us express Equation (17) in terms of the Rydberg unit of energy $\mathrm{Ry}=e^{2}/a_{B}\;=\;13.6\mathrm{eV}$ ($a_{B}=0.53\;Å$ is the Bohr radius):

$$N_{⊙}\approx \left(n_{⊙}\lambda_{0}\right)\frac{Z^{2}\mathrm{Ry}}{2k_{B}T\epsilon_{aq}}\left(\frac{a_{B}}{\lambda_{0}}\right)\frac{e^{-2\left(1+\frac{R_{0}}{\lambda_{0}}\right)}}{{\left(1+\frac{R_{0}}{\lambda_{0}}\right)}^{2}}∼10^{-2}\left(n_{⊙}\lambda_{0}\right).$$

Substituting Equation (17) into Equation (14) one finds

(18) $$\begin{array}{c} n_{⊙}^{max}=\frac{\epsilon_{aq}^{2}S_{\mathrm{tot}}}{2\pi eZ^{3}a_{B}}\left(\frac{\Delta T}{\Delta L}\right)\left(\frac{k_{B}T}{\mathrm{Ry}}\right)\times \\ e^{2\left(1+\frac{R_{0}}{\lambda_{0}}\right)}{\left(1+\frac{R_{0}}{\lambda_{0}}\right)}^{2}∼10^{15}{\mathrm{cm}}^{-3}. \end{array}$$

The maximal concentration of colloidal particles determined by Equation (18) corresponds to a volume fraction of $\phi^{min}\leq 0.001$ (see Figure 3). In Ref. [2], $\phi =0.006\to n_{⊙}≃5.45\;\cdot \;10^{15}$ cm ${}^{-3}$, thus it is easy to recalculate the estimation from Equation (18) $n_{⊙}^{max}∼10^{15}$ cm ${}^{-3}$$\to \phi^{min}∼0.01$, which surprisingly coincides well to the experimental findings of Ref. [2] (see Figure 3) considering the imperfect nature of metallic electrodes used in a real thermocell.

## 4. Conclusions

In this work we have studied the nontrivial role of colloidal particles in the formation of the Seebeck field in charged colloidal suspensions. The reasons for the two-stage character of the Seebeck effect observed in stabilized colloidal electrolytes are discussed. It is shown that the “initial state” is related to the phenomenon of thermal diffusion of the ions of the stabilizing electrolyte itself. We demonstrated that the presence of neutral colloids affects the Seebeck coefficient already in the initial state. This happens due to their influence on the polyelectrolyte conductivity and is consistent with the general understanding of the effective conductance of two-phase systems (see, e.g., [29]). As it was shown in Ref. [20] the presence in the bulk of stabilizing electrolyte of rarefied gas of colloids having a relatively large conductivity of the screening coats increases its effective conductivity. Accounting for this fact appears to explain the linear growth of the Seebeck coefficient as a function of the colloidal particles concentration observed in the experiment.

The ensuing steady state occurs when the thermodiffusion displacement of the colloidal particles becomes essential. The observed sharp drop in the Seebeck coefficient when the small concentration of the colloidal particles is added to the stabilizing electrolyte [2] is noteworthy. We propose an explanation of this feature basing on the specific behavior of colloidal particles in the vicinity of the charged electrode surface. Approaching the latter, a colloid loses its neutrality, discharging the accumulative layer of ions formed during the initial state. The decrease of the accumulative layer charge results in the drop of Seebeck signal. Our qualitative estimations give a surprisingly good correspondence to experimental findings.

It worth noting that importance of the DLVO–colloids’ interaction with the interface “electrolyte–conductor” was emphasized not only in the aged works of Wagner [15] and Onsager-Samaras [16]. The number of direct indications on the significance of such an interaction one can find also in the recent papers [30,31].

Finally, one can shed light on the discrepancy between the steady state establishment time lapse in the experiment ($\tau_{exp}∼8$ h) and the theoretical estimations counterpart by the authors of Ref. [2]. The latter, $\tau_{theor}={\left(\Delta L\right)}^{2}/D_{⊙}∼100$ h is calculated over the entire length scale of the thermocell. In the model developed here, on the other hand, the formation of mirror charges occurs in the close vicinity of the electrode/electrolyte interfaces. Furthermore, we wish to attract attention to the fact that due to the inhomogeneity of the charge density distribution along the thermocell length, the electric field also becomes non-homogeneous [22], resulting in the polarization of the colloidal particles and the acceleration of their motion with respect to a simple diffusion.

## References

[1] Dupont M.F., MacFarlane D.R., Pringle J.M. (2017). Thermo-electrochemical cells for waste heat harvesting—Progress and perspectives. Chem. Commun..

[2] Salez T.J., Huang B.T., Rietjens M., Bonetti M., Wiertel-Gasquet C., Roger M., Filomeno C.L., Dubois E., Perzynskic R., Nakamae S. (2017). Can charged colloidal particles increase the thermoelectric energy conversion efficiency?. Phys. Chem. Chem. Phys..

[3] Sehnem A.L., Janssen M. (2020). Determining single-ion Soret coefficients from the transient electrolyte Seebeck effect. arXiv.

[4] Riedl J.C., Akhavan Kazemi M.A., Cousin F., Dubois E., Fantini S., Lois S., Perzynski R., Peyre V. (2019). Colloidal Dispersions of Oxide Nanoparticles in Ionic Liquids: Elucidating the Key Parameters. ChemRxiv.

[5] Bacri J.C., Perzynski R., Salin D., Cabuil V., Massart R. (1990). Ionic ferrofluids: A crossing of chemistry and physics. J. Magn. Magn. Mater..

[6] Dubois E. (1999). Structural analogy between aqueous and oily magnetic fluids. J. Chem. Phys..

[7] Derjaguin B.V., Landau L.D. (1941). Theory of the stability of strongly charged lyophobic sols and of the adhesion of strongly charged particles in solutions of electrolytes. Acta Phys. Chem. URSS.

[8] Verwey E., Overbeek J. (1948). Theory of the Stability of Lyophobic Colloids.

[9] Landau L.D., Lifshitz E.M. (2011). Statistical Physics. Course of Theoretical Physics.

[10] Guthrie G., Wilson J.N., Schomaker V. (1941). Theory of the thermal diffusion of electrolytes in a Clusius column. J. Chem. Phys..

[11] Agar J.N., Turner J. (1960). Thermal diffusion in solutions of electrolytes. Proc. R. Soc. Lond. Ser. A.

[12] Agar J.N., Mou C.Y., Lin J.-I. (1989). Single-Ion Heat of Transport in Electrolyte Solutions: A Hydrodynamic Theory. Phys. Chem..

[13] Wurger A. (2010). Thermal non-equilibrium transport in colloids. Rep. Prog. Phys..

[14] Majee A., Wurger A. (2011). Collective thermo-electrophoresis of charged colloids. Phys. Rev..

[15] Wagner C. (1924). Die Oberflachenspannung verdunnter Elektrolytlosungen. Phys Z..

[16] Onsager L., Samaras N.J. (1934). The Surface Tension of Debye-Hückel Electrolytes. Chem. Phys..

[17] Kondepudi D., Prigogine I. (1999). Modern Thermodynamics: From Heat Engines to Dissipative Structures.

[18] Wurger A. (2008). Transport in Charged Colloids Driven by Thermoelectricity. Phys. Rev. Lett..

[19] Semenov S.N., Schimpf M.E. (2011). Thermodynamics of mass transport in diluted suspensions of charged particles in non-isothermal liquid electrolytes. C. R. Mec..

[20] Chikina I., Shikin V.B., Varlamov A.A. (2020). The Ohm law as alternative for the entropy origin nonlinearities in conductivity of dilute colloidal polyelectrolytes. Entropy.

[21] De Béthune A.J., Licht T.S., Swendeman N. (1959). The Temperature Coefficients of Electrode Potentials: The Isothermal and Thermal Coefficients—The Standard Ionic Entropy of Electrochemical Transport of the Hydrogen Ion. J. Electrochem. Soc..

[22] Chikina I., Shikin V.B., Varlamov A.A. (2012). Seebeck effect in electrolytes. Phys. Rev..

[23] Kohler W., Wiegand S. (2001). Thermal Nonequilibrium Phenomena in Fluid Mixtures.

[24] Piazza R., Guarino A. (2002). Soret Effect in Interacting Micellar Solutions. Phys. Rev. Lett..

[25] Demouchy G., Mezulis A., Bee A., Talbot D., Bacri J.C., Bourdon A. (2004). Diffusion and thermodiffusion studies in ferrofluids with a new two-dimensional forced Rayleigh-scattering technique. J. Phys..

[26] Chikina I., Shikin V.B., Varlamov A.A. (2015). Effect of boundary conditions on the character of ambipolar diffusion in electrolytes. Phys. Rev..

[27] Landau L.D., Lifshitz E.M. (2011). Electrodynamics of Continuous Media. Course of Theoretical Physics.

[28] Ohshima H. (1998). Electrostatic Interaction between a Sphere and a Planar Surface: Generalization of Point-Charge/Surface Image Interaction to Particle/Surface Image Interaction. J. Colloid Interface Sci..

[29] Landauer L. (1952). The Electrical Resistance of Binary Metallic Mixtures. J. Appl. Phys..

[30] Nadal F., Argoul F., Hanusse P., Pouligny B., Ajdari A. (2002). Electrically induced interactions between colloid particles in the vicinity of conducting plane. Phys. Rev..

[31] Palin M., Grier D., Han Y. (2007). Colloid electrostatic interactions near conducting surface. Phys. Rev..

